# Editorial: Exploring Novel Approaches to Eliminate HIV Reservoirs to Achieve a Cure for HIV

**DOI:** 10.3389/fcimb.2021.658848

**Published:** 2021-02-25

**Authors:** Renée M. van der Sluis, Andrés Finzi, Matthew S. Parsons

**Affiliations:** ^1^ Aarhus Institute of Advanced Studies (AIAS), Aarhus University, Aarhus, Denmark; ^2^ Department of Biomedicine, Aarhus University, Aarhus, Denmark; ^3^ Département de Microbiologie, Infectiologie et Immunologie, Université de Montréal, Montreal, QC, Canada; ^4^ Department of Microbiology and Immunology, McGill University, Montreal, QC, Canada; ^5^ Centre de Recherche du CHUM, Montreal, QC, Canada; ^6^ Yerkes National Primate Research Center, Emory University, Atlanta, GA, United States; ^7^ Department of Pathology and Laboratory Medicine, School of Medicine, Emory University, Atlanta, GA, United States

**Keywords:** human immunodeficiency virus (HIV), HIV cure, HIV cure research, HIV cure strategies, HIV latency

Antiretroviral therapy (ART) has revolutionized the treatment of HIV and has dramatically reduced morbidity and mortality among people living with HIV (PLWH) (Antiretroviral Therapy Cohort Collaboration, 2017). ART inhibits HIV replication, decreasing viral loads, halting disease progression and preventing sexual transmission of the virus (Attia et al., 2009; Volberding and Deeks, 2010; Cohen et al., 2011; Bavinton et al., 2018; Rodger et al., 2019). Cessation of ART, however, allows HIV to re-initiate replication (Davey et al., 1999; Colby et al., 2018). As such, ART is required throughout the lifespan of PLWH. Treatments that aim to cure HIV or induce viral remission are highly desirable to alleviate the need for lifelong ART.

The primary barrier to curing HIV is the persistence of a latent viral reservoir that predominantly resides in CD4^+^ T cells (Chun et al., 1997; Finzi et al., 1997; Wong et al., 1997). Numerous strategies have been proposed to target this reservoir (Pitman et al., 2018). Currently, the predominant strategy is the `shock and kill’ approach, which proposes employing latency reversing agents (LRAs) to induce virus expression and make virus harboring cells visible to the immune system. Subsequently, immune cells are engaged to eliminate cells harboring reactivated latent virus. Although clinical trials exploring the utility of the `shock and kill’ strategy have shown LRAs to induce viral expression in ART-treated PLWH, evidence of the efficacy of this approach for decreasing the frequency of cells carrying integrated HIV DNA has yet to be generated (Dufour et al., 2020). The reason(s) that `shock and kill’ strategies have not reduced the frequency of cells carrying integrated HIV DNA remains unclear. Novel LRAs and/or more potent anti-viral immune responses may be required to purge the virus (Kim et al., 2018; Dufour et al., 2020).

In this special issue of Frontiers in Cellular and Infection Microbiology, we invited early stage investigators to contribute their ideas as to how to tackle the important scientific problem of curing HIV infection. We received eight manuscripts, which we will briefly summarize to introduce this special issue.

Thomas et al. provide an excellent overview of recent advances towards understanding the latent viral reservoir and highlight various potential strategies for its eradication. To measure the success of these strategies, highly sensitive and accurate assays are required. Measuring the latent HIV reservoir is made difficult by variation in the viral genome, the low frequency of cells carrying latent virus and the presence of defective proviruses. Thomas et al. discuss the different methods that are available and debate the advantages and disadvantages of these assays.

Takahama and Yamamoto review the potential of using ligands for pattern recognition receptors (PRRs) as a cure strategy. PRR ligands can be used as vaccine adjuvants to enhance activation of the innate immune system and promote antigen-specific immune responses. PRR ligands can also be used to enhance preexisting immune responses. PRR ligands fit the ‘shock and kill’ approach well, because they can be used to activate the latent virus (i.e. deliver the “shock”) and boost the anti-viral immune response (i.e. the “kill”). Additionally, Takahama and Yamamoto discuss the possibility of combining PRR ligands with other immunotherapies, such as therapeutic vaccines, checkpoint inhibitors and broadly neutralizing antibodies (bNAbs).

Five of the articles in this special issue provide novel ideas as to how to enhance anti-viral immune responses through the use immunotherapeutic approaches.

Holder and Grant review the potential utility of immune checkpoint blockers targeting TIGIT to invigorate anti-viral immune responses. The advantage of using anti-TIGIT over other immune checkpoint blockers is that TIGIT is expressed on many HIV-specific CD8^+^ T cells in PLWH (Chew et al., 2016; Tauriainen et al., 2017) and blocking TIGIT can reinvigorate exhausted T cell responses. Additionally, anti-TIGIT therapy may also enhance NK cell function.

Mu et al. summarize recent developments pertaining to HIV-specific CAR T cells. One of the challenges Mu et al. describe is that CAR T cells need to function under conditions where low amounts of HIV-antigen are present. They discuss how combining CAR T cell therapy with LRAs might increase the effectiveness of this therapeutic option.

Juno and Kent summarize the different subsets of gamma-delta-(γδ) T cells and review their immunotherapeutic potential in HIV cure strategies. A major clinical advantage of employing these cells is the lack of MHC restriction for γδ T cell-mediated killing. This provides an opportunity for an “off-the-shelf” allogeneic product, circumventing the MHC-restriction present for most other cell-based immunotherapies.

van der Sluis et al. describe the potential of using plasmacytoid dendritic cells (pDCs) as an immunotherapy. PDCs are mostly known for their type I interferon-producing capacity, which can enhance NK cell activation and killing. However, pDCs can also improve T cell immunity by delivering antigens or therapeutic peptides combined with interferons.

Gardner provides an extensive overview of the potential of using recombinant Adeno-Associated Virus Vectors (rAAVs) to deliver bNAbs and engineered HIV inhibitors. Using rAAVs to deliver bNAbs or inhibitors would overcome the need for continued passive administration to sustain expression of these molecules in PLWH.

Finally, Ahlenstiel et al. summarize the recent advances toward developing an alternative HIV cure strategy, termed “block and lock.” This approach proposes to permanently silence HIV expression by “blocking” HIV transcription and “locking” the HIV promotor in a permanently latent state. Achievement of this premise would provide a functional cure, where a person still lives with HIV but the presence of the virus is not harmful in the absence of ART.

In summary, the articles presented in this special issue of Frontiers in Cellular and Infection Microbiology give an overview of the obstacles to developing successful HIV cure strategies. Additionally, the articles provide a collection of excellent novel ideas that could advance efforts to reduce the burden of integrated HIV DNA in ART-treated PLWH.

## Author Contributions

All authors contributed to the article and approved the submitted version.

## Funding

This project has received funding from the European Union’s Horizon 2020 research and innovation programme under the Marie Skłodowska-Curie grant agreement No 754513 and The Aarhus University Research Foundation (RMS).

## Conflict of Interest

The authors declare that the research was conducted in the absence of any commercial or financial relationships that could be construed as a potential conflict of interest.
